# Evaluation of prenatal central nervous system anomalies: obstetric management, fetal outcomes and chromosome abnormalities

**DOI:** 10.1186/s12884-022-04555-9

**Published:** 2022-03-15

**Authors:** Ann Gee Tan, Neha Sethi, Sofiah Sulaiman

**Affiliations:** Department of Obstetrics and Gynaecology, Faculty of Medicine, Universiti Malaya, 50603 Kuala Lumpur, Malaysia

**Keywords:** Prenatal ultrasound, Central nervous system anomalies, Chromosomal abnormality, Neurodevelopmental outcome

## Abstract

**Objective:**

To study the outcomes of fetuses who were diagnosed with central nervous system (CNS) anomalies during prenatal period and to describe the obstetric management of those pregnancies.

**Methods:**

In this retrospective study, fetuses who were detected to have central nervous system anomalies by prenatal ultrasound from January 2010 to December 2019 were recruited. Data regarding prenatal diagnosis and obstetric outcomes were retrieved from maternal and paediatric records. The prognosis of fetuses who were born alive was classified based on their neurodevelopmental outcome within two years of life.

**Results:**

There were a total of 365 fetuses with CNS anomalies within the 10-year study period, with a mean gestational age of 24.65±7.37 weeks at diagnosis. Ventriculomegaly (23.36%) was the commonest CNS anomalies seen. 198 (54.20%) of these fetuses had associated extra-CNS anomalies, with cardiovascular being the most common system involved. Fetal karyotyping was performed in 111 pregnancies, with chromosomal aberrations detected in 53 (49.07%) cases and culture failure in 3 cases. Majority of the chromosomal abnormalities were Edward syndrome (trisomy 18) and Patau syndrome (trisomy 13). Fetuses with congenital CNS anomalies and abnormal chromosomal karyotyping were more likely to be diagnosed earlier by prenatal ultrasound and tend to have poorer obstetric and neurocognitive prognosis. Prenatally, 86 (23.56%) of the cases were lost to follow up and likely to deliver elsewhere. Among the 279 cases whom their pregnancy outcomes were available, 139 (49.82%) pregnancies resulted in live births, 105 (37.63%) pregnancies were electively terminated, while the remaining 35 (12.54%) pregnancies ended in spontaneous loss. The decision of termination of pregnancy largely depends on mean diagnostic gestational age, presence of chromosomal aberrations and abnormal amniotic fluid volume in those fetuses. Two years after delivery, only 75 (53.96%) children out of 139 live births were still alive, 43 (30.93%) died and 21 (15.11%) cases were lost to follow-up. 32 (23.02%) children with prenatally diagnosed CNS anomalies had normal neurodevelopmental outcome. The presence of multiple CNS anomalies and involvement of extra-CNS anomalies indicated a poorer neurodevelopmental prognosis.

**Conclusion:**

Less than 50% of fetuses with prenatally diagnosed CNS anomalies resulted in live births. Even if they survive till delivery, 36.45% of them passed away within 2 years and 62.79% of children who survived till 2 years old had neurodevelopmental disability.

## Introduction

Congenital malformations are the leading factor of perinatal death and childhood morbidity [1–6]. Ultrasound screening has been a routine for all pregnant patients to detect structural anomalies [7–13], and it is recommended to perform this scan at 18-22 weeks of gestation [14–17].

Most of the time ultrasound (USG) screening provides reassurance to the couples, however, less than 5% of the scans revealed otherwise [4, 5, 17–19] CNS anomalies accounts for the highest percentage of prenatally detected deformities among all organ systems [3, 4, 11, 13, 20, 21], with an incidence of 2-10 per 1000 live births [22–25]. With the advancement in imaging technologies offering a better resolution of the fetal cerebral structures and enhancement in the skill of sonographers, the detection rate was expected to rise [14, 16, 26, 27].

Prenatal ultrasound and karyotyping findings often serve as the main guide for the prediction of prognostic outcomes and the establishment of management plans for fetuses with CNS anomalies. However, counseling on the prognosis is often difficult as most of the fetuses with these conditions ended up with abortion and neonatal death. There is also limited studies on long-term follow-up in children born with prenatally diagnosed CNS anomalies [7, 20]. Therefore, we intend to address the outcome of those fetuses and outline the systematic approach once CNS anomaly is suspected.

## Methodology

This was a retrospective cohort study that was performed at the Department of Obstetrics and Gynaecology, University of Malaya Medical Centre (UMMC) between January 2010 and December 2019. Pregnancies that were detected to have any CNS anomaly on ultrasonography at our institution within this ten-year duration were included.

UMMC was established as a tertiary referral center to receive referrals of pregnant patients with suspected congenital anomalies from all over Malaysia. All the prenatal ultrasound examinations were performed by sonographers or obstetricians and the prenatal diagnosis of CNS anomalies were confirmed by Maternal-Fetal Medicine specialists. All ultrasound examinations were performed using Voluson S8 (Buckinghamshire, United Kingdom) with 2- 5 MHz transabdominal transducer or 4-7 MHz transvaginal transducer. The obstetric USG assessment included a complete assessment of all morphological structures of the entire fetus. Any deformity noted from the scan was recorded according to respective organ systems. The fetus was considered to have multiple CNS anomalies if two or more CNS anomalies were revealed from prenatal scans. Fetus with both CNS and extra-CNS anomalies are classified to have multiple system deformities. Data collected also included gestational age (GA) at prenatal ultrasound diagnosis, maternal age, gravidity, parity, past obstetric and family history, sonographic findings on amniotic fluid volume and fetal parameters.

In our institution, cavum septi pellucidi (CSP) was considered wide when the transverse diameter was more than 10mm in thalamic view of the fetal head. Those fetuses with absent CSP without any other features suggestive of agenesis of corpus callosum were classified to have absent CSP. Cerebellar disorder in this study included cerebellar hypoplasia and/or dysplasia.

The abnormal sonographic findings were discussed among multidisciplinary teams to outline the management plan and establish the prognosis before parental counseling was carried out. Fetal karyotyping was recommended in all cases to determine any associated syndromic condition. Karyotype confirmation would be part of postnatal investigations.

After the multidisciplinary team meeting, TOP was an option offered to the couples if the anomaly potentially led to fetal or neonatal mortality and/or severe morbidity. TOP after 24 weeks was only offered for lethal and severely disabling abnormality, i.e. anencephaly. Post-mortem examination following elective and spontaneous abortions was recommended but rejected by all parents.

The maternal and infant records were traced to obtain the pregnancy outcomes. Fetuses with congenital CNS anomalies resulted in either termination of pregnancy (TOP), spontaneous abortion, stillbirth, or live birth. Live births were further grouped according to their survival durations.

Survivors in our study that were followed-up in our institution were evaluated via continuous neurodevelopmental assessments by pediatric neurologists and developmental pediatricians. Vision and hearing tests were done by ophthalmologists and Ear, Nose and Throat (ENT) physicians respectively. We reviewed their postnatal reports up to 2 years old or until they passed away, depending on which was earlier. Then, their neurocognitive functions were classified into normal development, motor delay, mental impairment, or motor and mental delay based on serial neurodevelopmental assessments performed by pediatricians. The neurodevelopmental assessment tool used in this study was created by Malaysian Paediatric Association and was used in all hospitals in Malaysia to detect and evaluate any developmental delay in gross motor, fine motor, social, speech and language. The child will be considered to have normal neurodevelopment if he/she passed the serial assessments as appropriate to the age and had not received any rehabilitative or neurotherapeutic treatment. Children with any significant motor or intellectual delay that was detected before they defaulted follow-up or passed away before 2 years old were classified as having abnormal development. Those with no neurological delay but defaulted follow-up or passed away before two years old were classified under the “lost to 2-year follow-up” category as we do not assume no abnormality until the age of two years.

### Statistics analysis

The data were analysed using SPSS, Version 12.0 (Statistical Package for Social Sciences, SPSS Inc., Chicago, IL, USA). The data were presented as percentages where appropriate. Pearson’s Chi-square test and t-test were used to test the differences between groups. The level of significance was set at *p* < 0.05.

## Result

We reported on 365 fetuses with abnormal central nervous system detected from prenatal ultrasound. There were two set of twins among them. The average maternal age was 31.26±5.13 years at the time of prenatal diagnosis. The diagnostic gestational age of fetuses ranged from 10 weeks to 39 weeks, with a mean of 24.65±7.37 weeks.

114 (31.40%) women were primigravida; whereas among the remaining 249 women, 90 (36.14%) of them experienced previous pregnancy loss and 226 (90.71%) women had at least one surviving child. There was a positive family history of CNS or chromosomal anomaly in eight women.

The prenatal ultrasonography represented 27 different anomalies that occurred 488 times in 365 fetuses and their distributions were described in Table 1. Overall, the commonest CNS anomaly detected on prenatal ultrasound was ventriculomegaly, which comprised 28 mild, 13 moderate, and 38 severe cases while the severity of the rest of 35 cases was unclassified. Based on the prenatal ultrasonographic findings, 198 (54.2%) had CNS anomalies that were associated with other systems’ anomalies, predominantly with cardiovascular system (20.37%), extremities (16.44%), and facial (15.51%) defects (Table 2). In the remaining 167 fetuses with isolated CNS anomalies, 33 (19.7%) of them were complicated with more than a single type of CNS anomalies. Anencephaly (87.80%) and cystic hygroma (53.33%) often occurred in isolation while microcephaly (85.71%), holoprosencephaly (82.85%), spinal malformations (82.76%), and agenesis of corpus callosum (81.25%) were predominantly associated with other systems’ anomalies (Table 1). Other anomalies were negligible due to insufficient data to make an inference.

**Table 1:** Distributions of 488 CNS anomalies in 365 fetuses

Types of CNS anomalies	Isolated CNS anomaly		Multiple system anomalies (n, %)	Total (n, %)
	Single CNS anomaly (n, %)	Multiple CNS anomaly (n, %)		
Neural tube defect				
- Anencephaly	36 (7.38)	1 (0.20)	4 (0.82)	41 (8.40)
- Encephalocele	9 (1.84)	6 (1.23)	9 (1.84)	24 (4.92)
-Meningocele	1 (0.20)	-	-	1 (0.20)
-Myelomeningocele	1 (0.20)	9 (1.84)	8 (1.64)	18 (3.69)
-Spina bifida occulta	-	-	1 (0.20)	1 (0.20)
-Unclassified closed spina bifida	1 (0.20)	-	-	1 (0.20)
-Unclassified NTD	1 (0.20)	6 (1.23)	5 (1.02)	12 (2.46)
Ventriculomegaly	29 (5.94)	24 (4.92)	61 (12.50)	114 (23.36)
Holoprosencephaly	5 (1.02)	1 (0.20)	29 (5.94)	35 (7.17)
Microcephaly	-	2 (0.41)	12 (2.46)	14 (2.87)
Cysts				
- arachnoid cyst	4 (0.82)	-	3 (0.61)	7 (1.43)
- choroid plexus cyst	1 (0.20)	-	10 (2.05)	11 (2.25)
Cystic hygroma	-	8 (1.64)	10 (2.05)	60 (12.30)
Chiari Type II malformation	-	-	2 (0.41)	18 (3.69)
Dandy Walker malformation	4 (0.82)	1 (0.20)	17 (3.48)	22 (4.51)
Cerebellar disorder	-	5 (1.02)	14 (2.87)	19 (3.89)
Megacisterna magna	1 (0.20)	4 (0.82)	18 (3.69)	23 (4.71)
Agenesis of corpus callosum	-	3 (0.61)	13 (2.66)	16 (3.28)
Other spinal malformations	4 (0.82)	1 (0.20)	24 (4.92)	29 (5.94)
Miscellaneous				
-sacrococcygeal teratoma	4 (0.82)	-	1 (0.20)	5 (1.02)
-cerebral atrophy	-	-	2 (0.41)	2 (0.41)
-wide CSP	-	-	3 (0.61)	3 (0.61)
-absent CSP	-	3 (0.61)	5 (1.02)	8 (1.64)
-megalencephaly	-	-	1 (0.20)	1 (0.20)
-hydranencephaly	-	-	1 (0.20)	1 (0.20)
-intracranial haemorrhage	-	1 (0.20)	-	1 (0.20)
- intracranial tumour	1 (0.20)	-	-	1 (0.20)

**Table 2:** Distributions of 432 extra-central nervous system anomalies in 198 fetuses

Organ systems	Frequency (n,%)
Cardiovascular	88 (20.37)
Extremities	71 (16.44)
Facial	67 (15.51)
Skull	55 (12.73)
Gastrointestinal	36 (8.33)
Musculoskeletal	33 (7.64)
Respiratory	33 (7.64)
Renal	27 (6.25)
Umbilicus	11 (2.55)
Placenta	4 (0.93)
Hepatobiliary	4 (0.93)
Genitourinary	3 (0.69)
Total	432 (100)

*CNS* central nervous system, *CSP* cavum septi pellucidi

Of the pregnancies, 67 of them were thought to have abnormal amount of amniotic fluid at the time of ultrasonography; polyhydramnios (*n*=41, 11.29%) appeared more frequently than oligohydramnios (*n*=26, 7.16%). Smaller than gestational age (growth parameters less than 10^th^ centile with normal and abnormal Doppler) occurred in 28 (7.67%) fetuses while 2 (0.55%) of anomalous fetuses were found to be larger than gestational age on prenatal ultrasonography.

Chromosomal analysis was attempted in 111 (30.41%) fetuses (Table 3). Of the karyotype analysis, 87 (78.38%) cases were performed prenatally via amniocentesis (98.20%)) and chorionic villous sampling (1.80%), whereas 24 (21.62%) cases were analyzed cytogenetically from neonatal tissue or blood samples. Ruling out three culture failures, abnormal karyotypes were recognized in 53 (49.07%) of 108 cases karyotyped. Edward syndrome (*n*=23, 43.40%) and Patau syndrome (*n*=14, 26.42%) were the commonest chromosomal aberrations in fetuses with CNS anomalies. Specific CNS anomaly shed light on the possibility of certain chromosomal abnormalities. Our study revealed a close relationship between holoprosencephaly with Patau syndrome, choroid plexus cyst with Edward syndrome, cystic hygroma with Turner syndrome, and megacisterna magna with Edward syndrome.

**Table 3:** Chromosomal abnormalities in 87 cases

Types of CNS anomalies	Normal karyotype (n, %)	Abnormal karyotype				
		Patau (n, %)	Edward (n, %)	Down (n, %)	Turner (n, %)	Miscellaneous (n, %)
*Neural tube defect*						
- Anencephaly	-	-	-	-	-	-
- Encephalocele	4 (4.60)	-	1 (1.15)	-	-	-
-Meningocele	1 (1.15)	-	-	-	-	-
-Myelomeningocele	2 (2.30)	1 (1.15)	-	-	-	-
-Spina bifida occulta	-	-	-	-	-	-
- Unclassified closed spina bifida	3 (3.45)	-	-	-	-	-
-Unclassified NTD	-	-	-	-	-	-
Ventriculomegaly	24 (27.59)	4 (4.60)	5 (5.75)	5 (5.75)	-	3 (3.45)
Holoprosencephaly	7 (8.05)	7 (8.05)	3 (3.45)	-	-	1 (1.15)
Microcephaly	2 (2.30)	2 (2.30)	-	1 (1.15)	-	-
*Cysts*						
- arachnoid cyst	1 (1.15)	1 (1.15)	-	-	-	-
- choroid plexus cyst	-	-	6 (6.90)	1 (1.15)	-	-
Cystic hygroma	7 (8.05)	1 (1.15)	-	-	5 (5.75)	-
Chiari Type II malformation	2 (2.30)	-	1 (1.15)	-	-	-
Dandy Walker malformation	5 (5.75)	4 (4.60)	4 (4.60)	-	-	-
Cerebellar disorder	5 (5.75)	1 (1.15)	-	1 (1.15)	-	-
Megacisterna magna	3 (3.45)	-	6 (6.90)	-	-	1 (1.15)
Agenesis of corpus callosum (ACC)	2 (2.30)	1 (1.15)	-	1 (1.15)	-	-
Other spinal malformations	7 (8.05)	1 (1.15)	-	-	-	-
*Miscellaneous*						
-sacrococcygeal teratoma	-	-	-	-	-	-
-cerebral atrophy	-	-	-	-	-	-
-wide CSP	-	-	1 (1.15)	-	-	-
-absent CSP	1 (1.15)	-	2 (2.30)	-	-	-
-megalencephaly	-	-	-	-	-	-
-hydranencephaly	-	-	-	-	-	-
-intracranial haemorrhage	-	-	-	-	-	-
-intracranial tumour	-	-	-	-	-	-

*CNS* central nervous system, *CSP* cavum septi pellucidi

We identified 279 fetuses for whom the obstetric outcomes could be found from retrospective tracing (Table 4). The survival of anomalous fetuses was only achieved in 139 (49.82%) due to the high rate of induced TOP (*n*=105, 37.63%) with additional 35 (12.54%) spontaneous fetal demise (8 intrauterine fetal death; 27 stillbirths). Moreover, 36 (25.90%) of the neonates died in the first month after birth, 3 (2.16%) of them survived till less than 6 months in life and 4 (2.88%) infants were alive for more than 6 months but less than a year. Only 75 (53.96%) live births remained alive after 2 years since birth. 21 (15.11%) live births were lost to follow-up, thus their survival durations remained undetermined.

**Table 4:** Obstetric outcomes in 279 fetuses

Types of CNS anomalies	TOP		IUFD	SB	LB					
	(<24 wks)	(>24 wks)			<1 mth survival	1 mth to <6 mths survival	6 mths< 1 yr survival	1-2 yrs survival	>2 yrssurvival	Lost to follow-up
*Neural tube defect*										
- Anencephaly	22	9	-	2	-	3	-	-	-	-
- Encephalocele	10	3	-	2	-	-	-	-	3	1
-Meningocele	-	-	-	-	-	-	-	-	1	-
-Myelomeningocele	3	-	-	1	3	-	-	-	6	3
-Spina bifida occulta	-	-	-	-	-	-	-	-	1	-
- Unclassified closed spina bifida	-	-	-	-	-	-	-	-	1	-
-Unclassified NTD	2	2	-	1	1	-	-	-	6	-
Ventriculomegaly	4	8	-	7	14	3	4	-	45	11
Holoprosencephaly	6	4	1	2	8	-	-	-	3	1
Microcephaly	2	1	-	1	4	-	-	-	-	1
*Cysts*										
- arachnoid cyst	-	-	-	2	1	-	-	-	2	-
- choroid plexus cyst	2	1	-	1	1	-	-	-	2	-
Cystic hygroma	18	1	6	3	1	-	-	-	8	3
Chiari Type II malformation	3	3	-	2	1	-	-	-	7	1
Dandy Walker malformation	-	3	-	5	4	-	-	-	4	1
Cerebellar disorder	-	2	1	2	3	2	1	-	3	-
Megacisterna magna	-	2	-	2	2	1	-	-	4	1
Agenesis of corpus callosum	3	4	-	-	2	-	1	-	3	1
Other spinal malformations	8	4	-	3	5	-	-	-	1	2
*Miscellaneous*										
-sacrococcygeal teratoma	1	-	-	-	-	-	-	-	2	1
-cerebral atrophy	-	-	-	-	-	1	-	-	1	-
-wide CSP	-	-	-	1	-	-	-	-	1	-
-absent CSP	1	-	-	-	1	-	-	-	3	-
-megalencephaly	-	-	-	-	-	-	-	-	1	-
-hydranencephaly	-	-	-	-	1	-	-	-	-	-
-intracranial haemorrhage	-	-	-	-	1	-	-	-	-	-
-intracranial tumour	-	-	-	-	-	-	-	-	-	-

*CNS* central nervous system, *CSP* cavum septi pellucidi, *SB* stillbirth, *LB* live birth, *wks* weeks, *mths* months

Follow-up data on delivery methods were available for 257 fetuses involved in the study. The fetuses were delivered at the average GA of 30.90±8.49 weeks. In specific, the mean GA for elective TOP, intra-uterine fetal loss, and live births were 21.93±5.99 weeks, 30.71±7.32 weeks, and 37.15±2.71 weeks respectively. In 124 cases of elective and spontaneous termination of pregnancy, 115 (92.74%) cases were performed vaginally via medical induction and 9 (7.26%) cases required surgical evacuation on the conception. On the other hand, live births were delivered more frequently through Caesarean section (*n*=78, 58.65%) than vaginal delivery (*n*=55, 41.35%).

139 surviving infants were classified according to their respective functional status based on two-year neurodevelopmental assessments (Table 5). Normal neurologic development was achieved in 32 (23.02%) children. Functionally abnormal children included 14 (10.07%) cases with pure motor disability, 9 (6.47%) cases with pure mental disability as well as 31 (22.30%) children with both motor and intellectual impairments. The remaining 53 (38.13%) children did not have any significant abnormality detected before they defaulted neurodevelopmental follow-up or passed away before 2 years old, therefore classified as “lost to follow-up” in Table 5. Among those children with neurological deficit, 17 (31.48%) of them had complete paralyzation, 11 (20.37%) of them experienced bladder and/or bowel incontinence, 7 (12.96%) of them had concomitant psychiatric disorder, 7 (12.96%) of them suffered from epilepsy, 6 (11.11%) of them had hearing loss, and 12 (22.22%) of them were reported to have vision problems.

**Table 5:** Two-year neurodevelopmental outcomes of 139 surviving infants

Types of CNS anomalies	Normal Development	Motor disability	Mental disability	Psychomotor disability	Lost to follow-up
*Neural tube defect*					
- Anencephaly	-	-	-	-	3
- Encephalocele	-	1	1	2	-
-Meningocele	-	-	-	1	-
-Myelomeningocele	1	3	-	3	5
-Spina bifida occulta	1	-	-	-	-
- Unclassified closed spina bifida	-	1	-	-	-
-Unclassified NTD	2	3	-	1	1
Ventriculomegaly	16	9	7	19	26
Holoprosencephaly	-	2	-	2	8
Microcephaly	-	-	-	1	4
*Cysts*					
- arachnoid cyst	2	-	-	-	1
- choroid plexus cyst	1	-	-	1	1
Cystic hygroma	8	-	-	2	2
Chiari Type II malformation	1	3	-	3	2
Dandy Walker malformation	2	-	1	1	5
Cerebellar disorder	-	-	-	3	6
Megacisterna magna	1	1	-	3	3
Agenesis of corpus callosum	-	2	-	3	2
Other spinal malformations	1	-	-	1	6
*Miscellaneous*					
-sacrococcygeal teratoma	1	1	-	-	1
-cerebral atrophy	-	-	-	1	1
-wide CSP	-	-	-	1	-
-absent CSP	-	-	-	3	1
-megalencephaly	-	-	-	1	-
-hydranencephaly	-	-	-	-	1
-intracranial haemorrhage	-	-	-	-	1
-intracranial tumour	-	-	-	-	-

*CNS* central nervous system, *CSP* cavum septi pellu

## Discussion

Hadzagic et al. [8] defined congenital malformation as “any morphological abnormality that dates to the embryonic or fetal period, regardless of the mechanism of its origin “. As radiological technology advances, fetal ultrasonography is an appropriate diagnostic standard for prenatal CNS anomalies and they can be visualized starting from the first trimester, even as early as 6 weeks onwards [7, 17, 27–29]. The detection of CNS anomaly on ultrasound requires a systemic approach to direct into the final diagnosis, evaluate the prognosis of the condition and outline the management plan accordingly.

### Fetal magnetic resonance imaging (fMRI)

There is a concern that under/overdiagnosis might affect the decision on management options and lead to inappropriate TOP [26], thus the prenatal diagnosis established must be precise. Whenever the prenatal prediction by USG is uncertain, fMRI can be performed in addition to USG to secure the diagnosis [7, 8, 30]. However, only six (1.65%) pregnant patients decided to proceed with fMRI after detection of central nervous system anomaly from prenatal ultrasound. In this cohort, fMRI provided additional information and/or corrected misinterpreted ultrasound findings in five of the cases.

### Multidisciplinary involvement in genetic counseling

The obstetric management of these pregnancies would be individualised based on the prognosis of the fetus and maternal risk factors. The management is complex, and the women are best cared for by a multidisciplinary team involving feto-maternal specialist, neonatologist, paediatrics neurosurgeons and geneticists [7, 11, 14, 30, 31]. Geneticists should be involved to evaluate the possibility of the condition being hereditary in nature and assess the risk of recurrence in future pregnancies [14, 30–32]. Once the management outline is established, genetic counseling with prospective parents is mandatory to provide them the consultant information on the specific disorder [32–35]. Joint discussion among clinicians and families has been proven to aid the parents in decision-making [30, 32, 36]. However, the process should be non-directive and the counselors should maintain a neutral position to respect for couples’ autonomy. Faced with the diagnosis of CNS disorder, the couples are usually unprepared and breaking this bad news inevitably evokes psychological stress as they are forced to confront the reality, especially when they are considering to end the pregnancy which can be precious to them [33, 37]. Continuation of pregnancy and if the child survives, he/she will be further assessed on the neurodevelopment by a team of neurologists, neonatologists and pediatricians [30]. Collaboration with bereavement counselors and social workers will favor the process.

Following the discussion, parents will be given few options. They need to decide whether to proceed to further invasive testing which may be diagnostic, as well as decisions about whether to give their fetuses a chance to live but possibly suffer from morbidity or terminating the pregnancy to avoid the risk of suffering [30, 33]. Parents should be informed of the risk of their decision made and the possibility of false-positive prenatal diagnosis before they decide to proceed with any therapeutic intervention [9, 30]. The time to make decisions after the counselling is quite limited. The involvement of multidisciplinary team is crucial as it would aid parents in making decision.

### Factors affecting TOP

Parents are often faced with the difficulty weighing the severity and survival of the condition [34], and their decision on TOP is affected by various factors [7, 33, 38, 39]. When the CNS anomalies were diagnosed before viability period of 24 weeks, 62.38% of them decided to terminate the pregnancy; whereas 84.68% of the pregnancies diagnosed after 24 weeks were put under conservative management. The GA at prenatal diagnosis significantly affected the decision for TOP (p <0.001). There is an increasing incidence of TOP when chromosomal and amniotic fluid abnormalities are present, with significant p-values of <0.001 and 0.017 respectively. Maternal age, presence of children, previous uncompleted pregnancies, involvement of additional CNS and extra-CNS anomalies do not significantly influence the consideration of TOP (p=0.526, 0.981, 0.579, 0.124, 0.936 respectively).

### Very late termination of pregnancy (VLTOP)

The parental decision-making process takes time [7, 40], and our patients took an average of 1.83±2.20 weeks to decide on TOP after the prenatal diagnoses were made. In addition, majority (*n*=203, 55.61%) of the diagnoses were made only after 24 weeks gestation. Therefore, VLTOP seems to be unavoidable [7, 40, 41]. It has been shown that CNS anomalies have the highest percentage leading to VLTOP among all systems [40]. VLTOP is a subject of ethical debate as TOP after 24 weeks gestation is not accepted in many countries [8, 33, 40]. In contrast, Malaysia has made TOP after 24 weeks legally permissible. Abortion Act 1967 has stated that TOP at any GA is allowed when “there is a substantial risk that if the child were born, he would suffer from such mental or physical abnormalities as to be seriously handicapped”.

### Conservative management

When the couples decide on conservative management, the clinicians have the responsibility to inform them about the predicted prognosis and possible neurodevelopmental outcome to reduce anxiety when the affected child is delivered. Serial sonographic assessments are crucial to monitor any progression of the anomaly that might revise the prognosis as pregnancy advances [42].

### In-utero surgery

Although none of the women in our study was offered for in-utero surgery as part of obstetric management, there are various publications that showed that many CNS anomalies are surgically treatable by fetal therapeutic surgery [11, 43, 44]. Rapidly advancing Fetal Medicine will soon make fetal surgery a capable modality for the correction of some fetal malformations.

### In-utero fetal demise

Even when some parents decide to continue the pregnancy or could not make up their minds for TOP, many of these fetuses with CNS anomalies are incompatible with life and would end up with spontaneous fetal demise [19, 20, 45]. We found an association between the involvement of multiple systems and higher rates of in-utero fetal demise with a significant *p*-value of 0.001.

### Infectious etiology

Although the underlying cause of central nervous system anomalies remains obscure in many cases [2, 5, 7, 8, 46], effort should be put in to establish the aetiology that can revise the recurrence risk. Infectious screening is mandatory to exclude TORCH (toxoplasma, rubella, cytomegalovirus, herpes simplex, and syphilis) exposure during the prenatal period [7, 30, 47].

### Chromosomal anomaly

Fetal karyotyping should be part of obstetric practices to exclude any chromosomal anomaly [30, 47–49], and if it fails, postnatal cytogenic analysis should be carried out [7]. An abnormal karyotype was more often present in older women (30.44 years versus 32.26 years, *p*=0.074) and when multiple anatomic systems are involved (*p*<0.001). Fetuses with chromosomal anomalies were more likely to be diagnosed earlier (24.6818 weeks versus 26.25 weeks, *p*=0.231) and commonly illustrate fetal growth restriction on serial growth ultrasonography (*p*=0.002). In continuing pregnancies with chromosomal anomalies, the obstetric outcome was anticipated to be poorer in most cases, with higher rates of induced (*p*<0.001) and spontaneous (*p*=0.825) loss of pregnancy. In cases which the fetuses remained viable till delivery, the child was expected to have a higher probability of being neurocognitively abnormal, although the association was not statistically significant due to the small sample size (*p*=0.901).

### Mode of delivery

Maternal well-being is always the priority for clinicians and the pregnancy should not be continued if severe maternal complications are anticipated. Vaginal delivery was aimed for fetuses with poor prognosis as Caesarean section might complicate subsequent pregnancies. In our study, parents who decided for termination of pregnancy were more often being delivered vaginally. On the contrary, live births were more commonly being delivered via Caesarean section (*p*<0.001) as complications like cephalopelvic disproportion and rupture of cystic lesion were foreseen in pregnancies complicated with CNS anomalies [42, 50].

### Post-mortem investigations

In any pregnancy that resulted in fetal loss, be it induced, or in-utero demise, complete post-mortem investigation should not be neglected [7, 10, 11, 26, 30]. In these cases, autopsy should be recommended to be part of the post-mortem examination to verify and revise the prenatal diagnosis which can provide beneficial information for subsequent pregnancies [11, 14, 26]. However, in our study, all parents were reluctant to proceed with the pathological examination contrary to the high autopsy rates in other countries [51–54]. Given this limitation, magnetic resonance imaging (MRI) had been suggested as a useful adjunct and increasing in popularity. Recent reviews had shown that post-mortem MRI was as effective as autopsy and advocated to replace autopsy in reaching the postnatal confirmatory diagnosis when autopsy was denied [14, 55–57]. When post-mortem autopsy or MRI was not feasible, a thorough gross examination by neonatologists should be performed and photographs of the fetus should be captured for diagnostic purposes [58].

### Live births

Those fetuses that survive till delivery, further investigations such as cranial ultrasound and MRI of brain and spine should be ordered to confirm the prenatal prediction of the CNS anomaly. The child should have long-term neurodevelopment follow-up under paediatric neurologists and paediatricians. Hearing and visual assessments should be done before the child was discharged home. Speech therapists, physiotherapists, and occupational therapists should be involved if the child suffers from any neurological deficit [30]. Most of the children would need to go through at least one surgery to correct CNS defects after birth [30, 59, 60].

### Neurodevelopmental outcomes

The prognostic outcome of children often dependent on the severity of anomalies [7], therefore distinguishing the severity of involvement by fetal ultrasonography is of paramount importance. For example, in cases of ventriculomegaly, 80% (*n*=20/25) of neurodevelopmentally abnormal children were detected prenatally to have moderate and severe ventriculomegaly while 57.14% (*n*=8/14) of fetuses being diagnosed with mild ventriculomegaly had normal paediatric examinations, *p* = 0.018. The overall prognosis also strongly depends on the presence of multiple CNS anomalies and involvement of other systems. Fetuses with isolated anomaly and single system anomaly indicated favourable postnatal outcomes as compared to those with multiple CNS anomalies and involvement of multiple organ systems (*p*=0.006, *p*=0.006 respectively).

### Strength

Prognosticating the long-term neurodevelopmental outcome in children who had been diagnosed prenatally with CNS anomalies remains a challenge as prenatal diagnostic tools could not determine the functional status and there is limited research on this aspect [30, 33]. Our study that comprehensively scrutinizes the impact of prenatal CNS anomalies on postnatal life up to 2 years added to literature the useful prognostic information that was often sought by the clinicians and parents during genetic counseling. To the best of our knowledge, this is the largest cohort on prenatal CNS anomalies for live births and dead fetuses with wide spectrums of anomalies.

### Limitations

We acknowledge that many patients in our study were lost to follow-up, and the neurodevelopmental assessment of the survivors was heterogeneous and performed by different paediatricians with different standards of protocols.

## Conclusion

In the present study, the survival rate was only 49.82% due to the high rate of TOP and in-utero demise, and major portions of the surviving children were moderately or severely disabled. With advanced development in Fetal Medicine, more structurally abnormal fetuses could survive but they are at risk of morbidity throughout their life which greatly impact their families, healthcare systems as well as societies [1, 2, 4, 19, 21]. Primary prevention plays a big role especially in the reduction of the incidence of neural tube defect [3, 7, 8, 20, 61], and secondary prevention by routine USG examination to all pregnant patients should remain the mainstay of obstetrical screening [1, 3, 8, 40].

## Data Availability

The datasets used and/or analysed during the current study are available from the corresponding author on reasonable request.

## Abbreviations

CNS: central nervous system
USG: ultrasound
UMMC: University of Malaya Medical Centre
GA: gestational age
TOP: termination of pregnancy
ENT: Eye, Nose, Throat
VLTOP: very late termination of pregnancy
TORCH: toxoplasma, rubella, cytomegalovirus, herpes simplex, and syphilis
MRI: magnetic resonance imaging
fMRI: fetal magnetic resonance imaging
CSP: cavum septi pellucidi
SB: stillbirth
LB: live birth
